# Impact of new-onset atrial fibrillation on 12-month outcomes and mortality after esophagectomy

**DOI:** 10.1093/dote/doag058

**Published:** 2026-06-22

**Authors:** Hani Essa, Ashwin Balu, Brian Johnston, Nathan Stephens, Gregory Y H Lip, Ingeborg Welters

**Affiliations:** Department of Cardiovascular and Metabolic Medicine, Institute of Life Course and Medical Sciences, University of Liverpool, Liverpool, UK; Royal Liverpool Intensive Care Unit, University Hospitals of Liverpool Group, Liverpool, UK; Liverpool Centre for Cardiovascular Science, University of Liverpool, Liverpool John Moores University and Liverpool Heart and Chest Hospital, Liverpool, UK; Department of Cardiovascular and Metabolic Medicine, Institute of Life Course and Medical Sciences, University of Liverpool, Liverpool, UK; Royal Liverpool Intensive Care Unit, University Hospitals of Liverpool Group, Liverpool, UK; Liverpool Centre for Cardiovascular Science, University of Liverpool, Liverpool John Moores University and Liverpool Heart and Chest Hospital, Liverpool, UK; Department of Cardiovascular and Metabolic Medicine, Institute of Life Course and Medical Sciences, University of Liverpool, Liverpool, UK; Royal Liverpool Intensive Care Unit, University Hospitals of Liverpool Group, Liverpool, UK; Liverpool Centre for Cardiovascular Science, University of Liverpool, Liverpool John Moores University and Liverpool Heart and Chest Hospital, Liverpool, UK; Department of Upper Gastrointestinal Surgery, Royal Liverpool University Hospital, Liverpool, UK; Liverpool Centre for Cardiovascular Science, University of Liverpool, Liverpool John Moores University and Liverpool Heart and Chest Hospital, Liverpool, UK; Department of Clinical Medicine, Aalborg University, Aalborg, Denmark; Department of Cardiology, Lipidology and Internal Medicine, Medical University of Bialystok, Bialystok, Poland; Department of Cardiovascular and Metabolic Medicine, Institute of Life Course and Medical Sciences, University of Liverpool, Liverpool, UK; Royal Liverpool Intensive Care Unit, University Hospitals of Liverpool Group, Liverpool, UK; Liverpool Centre for Cardiovascular Science, University of Liverpool, Liverpool John Moores University and Liverpool Heart and Chest Hospital, Liverpool, UK

**Keywords:** atrial fibrillation, cardiovascular outcomes, esophagectomy, esophageal cancer, postoperative complications

## Abstract

New-onset atrial fibrillation (NOAF) is a frequent complication after esophagectomy. While associated with adverse short-term outcomes, its longer-term cardiovascular impact remains uncertain. We evaluated associations between NOAF and postoperative cardiovascular and respiratory complications using a large international real-world dataset. We performed a retrospective cohort study using the TriNetX global federated health research network. Adults undergoing esophagectomy were identified using ICD-10, CPT, and SNOMED codes, excluding those with prior atrial fibrillation or flutter. NOAF was defined within 30 days postoperatively. Propensity score matching (1:1) balanced demographics, comorbidities, and medication use. Outcomes were assessed over 12 months using Cox proportional hazards models. We analyzed 1977 matched patients (mean age 67.1 ± 8.7 years; 18% female). Within 0–3 months, NOAF was associated with increased risks of death (HR 2.09, 95% CI 1.62–2.70), heart failure (HR 2.03, 95% CI 1.32–3.11), myocardial infarction (HR 2.35, 95% CI 1.17–4.74), pneumonia (HR 1.80, 95% CI 1.42–2.29), venous thromboembolism (HR 1.76, 95% CI 1.17–2.66), and rehospitalization (HR 2.78, 95% CI 2.42–3.20), with no significant association for stroke. Between 3 and 12 months, elevated risks persisted for death (HR 1.60, 95% CI 1.30–1.98) and heart failure (HR 2.55, 95% CI 1.37–4.73). Across 12 months, NOAF remained associated with death (HR 1.76, 95% CI 1.50–2.07) and heart failure (HR 2.23, 95% CI 1.56–3.19). NOAF after esophagectomy identifies patients at increased risk of major postoperative and cardiovascular complications, particularly early after surgery.

## Graphical Abstract

**Graphical Abstract:**
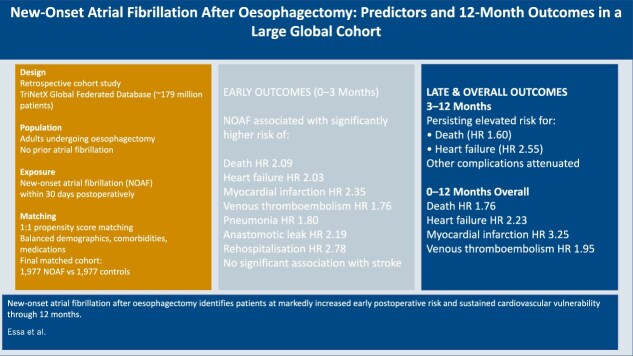

## INTRODUCTION

Esophageal cancer ranks as the eighth most commonly diagnosed malignancy and the sixth leading cause of cancer-related mortality worldwide.^1^ The cornerstone of curative treatment is esophagectomy, typically combined with perioperative chemotherapy, radiotherapy, or immunotherapy depending on histological subtype. Despite advances in surgical techniques and perioperative care, esophagectomy is associated with significant morbidity, with complication rates reported in up to ~60% of patients.^2^

Postoperative complications include pulmonary infections, anastomotic leak, and cardiovascular events, all of which contribute to prolonged hospitalization and increased mortality. Among these, new-onset atrial fibrillation (NOAF) is the most commonly encountered arrhythmia post esophagectomy, likely due to extensive surgical dissection, intrathoracic manipulation, and physiological stress associated with this operation.^3^^,^^4^ Among patients undergoing esophagectomy without a prior history of atrial fibrillation (AF), NOAF occurs in approximately 16.5% of cases and may be associated with a prolonged hospitalization and increased perioperative mortality.^5^

As postoperative and oncologic outcomes continue to improve with advances in surgical and adjuvant therapies,^6^ understanding the long-term cardiovascular implications of NOAF and its association with surgical complications and infections in the postoperative period has become increasingly important. Leveraging the large, multicenter, real-world data available through a global federated research network, this study aimed to evaluate the association between NOAF and long-term mortality and cardiovascular outcomes following esophagectomy.

## METHODS

We conducted a retrospective analysis using the TriNetX global health research network, which aggregates deidentified electronic health record data from approximately 179 million patients. The network provides access to deidentified electronic health record data and enables the creation of patient cohorts based on diagnostic and procedural codes. Analyses are conducted in a privacy-preserving manner, with only aggregated results available to investigators. The database complies with major data protection regulations including Health Insurance Portability and Accountability Act and General Data Protection Regulation.^7^

### Ethics statement

This study utilized deidentified data obtained from the TriNetX global health research network. Each participating Healthcare Organization contributes data in compliance with its own ethical and legal obligations, including patient consent and institutional approvals, as governed by a Business Associate Agreement. Because the dataset contains only anonymized information used exclusively for research purposes, the study did not meet the definition of human subjects’ research and thus did not require formal ethics committee approval.

### Study design

The search was conducted on 9 November 2025, with predefined inclusion and exclusion criteria based on the relevant International Classification of Diseases, Tenth Revision, Clinical Modification codes (ICD-10) to create our cohort. All patients undergoing esophagectomy for esophageal cancer were included. The TriNetX database was searched for results between 1 January 2000 and 1 January 2025. Eligible patients were identified and then two cohorts created based on the presence of NOAF and atrial flutter recorded in a patient’s medical records in the first 30 days post-esophageal resection. Patients were excluded if they had AF recorded in their medical notes prior to their operation.

### Statistical analysis

Data were analyzed relative to the index event, defined as the date of esophagectomy, with a 12-month follow-up period. Continuous variables were summarized as mean (standard deviation, SD).

Propensity score matching (PSM) was performed to reduce baseline differences between patients with and without NOAF. Patients were matched 1:1 using a logistic regression model incorporating baseline characteristics and medication use (detailed in Table 1). Matching was conducted using nearest-neighbor methodology with a caliper of 0.1, and covariate balance was assessed using standardized mean differences (<0.1 considered acceptable). Time-to-event outcomes were analyzed using Cox proportional hazards models, with results reported as hazard ratios (HRs) and 95% confidence intervals (CIs). Patients were censored at death, last follow-up, or 12 months.

**Table 1:** Baseline demographic, clinical, laboratory, and pharmacologic characteristics of patients with and without new-onset atrial fibrillation AF before and after propensity score matching (PSM)

**Subfeature**	**Before matching (new-onset AF)**	**Before matching (control)**	***P*-value (BM)**	**Std diff. (BM)**	**After matching (new-onset AF)**	**After matching (control)**	***P*-value**	**Std difference**
Age at index (years)	67.1 ± 8.66	56.3 ± 14.5	<0.001	0.91	67.1 ± 8.66	67.5 ± 9.47	0.74	0
White	1579 (79.87%)	20,593 (30.97%)	<0.001	1.01	1579 (79.87%)	1545 (78.15%)	0.13	0.04
Female	343 (17.35%)	36,835 (55.39%)	<0.001	0.81	343 (17.35%)	329 (16.64%)	0.26	0.03
Black or African American	66 (3.34%)	802 (1.21%)	<0.001	0.12	66 (3.34%)	69 (3.49%)	1	<0.01
Asian	36 (1.82%)	1380 (2.08%)	0.14	0.03	36 (1.82%)	40 (2.02%)	0.17	0.04
Hispanic or Latino	21 (1.06%)	400 (0.60%)	0.01	0.05	21 (1.06%)	29 (1.47%)	0.15	0.04
Hypertension	1009 (51.04%)	8421 (12.66%)	<0.001	0.85	1009 (51.04%)	1046 (52.91%)	0.4	0.02
Ischemic heart disease	478 (24.18%)	2738 (4.12%)	<0.001	0.54	478 (24.18%)	472 (23.88%)	0.48	0.02
Diabetes mellitus	389 (19.68%)	2361 (3.55%)	<0.001	0.5	389 (19.68%)	383 (19.37%)	0.91	0
Obesity	271 (13.71%)	2234 (3.36%)	<0.001	0.38	271 (13.71%)	265 (13.40%)	0.9	0
COPD	244 (12.34%)	1088 (1.64%)	<0.001	0.38	244 (12.34%)	225 (11.38%)	0.26	0.03
Cerebrovascular disease	140 (7.08%)	1014 (1.53%)	<0.001	0.25	140 (7.08%)	115 (5.82%)	0.59	0.02
Chronic kidney disease	99 (5.01%)	741 (1.11%)	<0.001	0.22	99 (5.01%)	121 (6.12%)	0.95	0
Heart failure	94 (4.76%)	523 (0.79%)	<0.001	0.24	94 (4.76%)	92 (4.65%)	0.89	0
Peripheral vascular disease	84 (4.25%)	299 (0.45%)	<0.001	0.25	84 (4.25%)	77 (3.90%)	0.23	0.03
Pulmonary embolism	66 (3.34%)	350 (0.53%)	<0.001	0.2	66 (3.34%)	64 (3.24%)	0.87	0
Deep vein thrombosis	31 (1.57%)	142 (0.21%)	<0.001	0.15	31 (1.57%)	35 (1.77%)	0.59	0.02
Tobacco use	109 (5.51%)	694 (1.04%)	<0.001	0.22	109 (5.51%)	106 (5.36%)	0.66	0.01
Hemoglobin A1c (%)	6.09 ± 1.24	5.61 ± 1.04	<0.001	0.4	6.09 ± 1.24	5.97 ± 1.16	0.18	0.09
Proton pump inhibitors	958 (48.46%)	14,425 (21.69%)	<0.001	0.71	958 (48.46%)	973 (49.22%)	0.29	0.03
Beta blockers	695 (35.15%)	4697 (7.06%)	<0.001	0.8	695 (35.15%)	673 (34.04%)	0.65	0.01
Aspirin	436 (22.05%)	2988 (4.49%)	<0.001	0.58	436 (22.05%)	456 (23.07%)	0.59	0.02
Clopidogrel	100 (5.06%)	1041 (1.57%)	<0.001	0.22	100 (5.06%)	107 (5.41%)	0.53	0.02

Values are presented as mean ± standard deviation (SD) for continuous variables and *n* (%) for categorical variables. ‘AF-free’ refers to patients without AF at index, and ‘AF group’ refers to those who developed AF. Comparisons before PSM represent the unmatched cohorts, and those after PSM represent the matched cohorts. *P* values <0.05 were considered statistically significant. Diagnostic and medication categories correspond to ICD-10-CM and ATC/RxNorm classifications, respectively. COPD, chronic obstructive pulmonary disease; SSMD , standardized mean difference; SGLT2, sodium–glucose cotransporter 2.

Outcomes included all-cause mortality, myocardial infarction, stroke, heart failure, venous thromboembolism, rehospitalization, and postoperative complications. Patients with prior history of a given outcome were excluded from its respective analysis. Time-to-event outcomes were analyzed using Cox proportional hazards models, with results reported as HRs and 95% CIs. *E*-values were calculated to assess the impact of unmeasured confounding. A two-tailed *P*-value <0.05 was considered statistically significant. Analyses were performed using R (version 4.3.1).^8^  *E*-values were calculated to quantify the sensitivity of the observed associations to potential unmeasured confounding. A two-tailed *P*-value <0.05 was considered statistically significant.^9^

## RESULTS

**Fig. 1:**
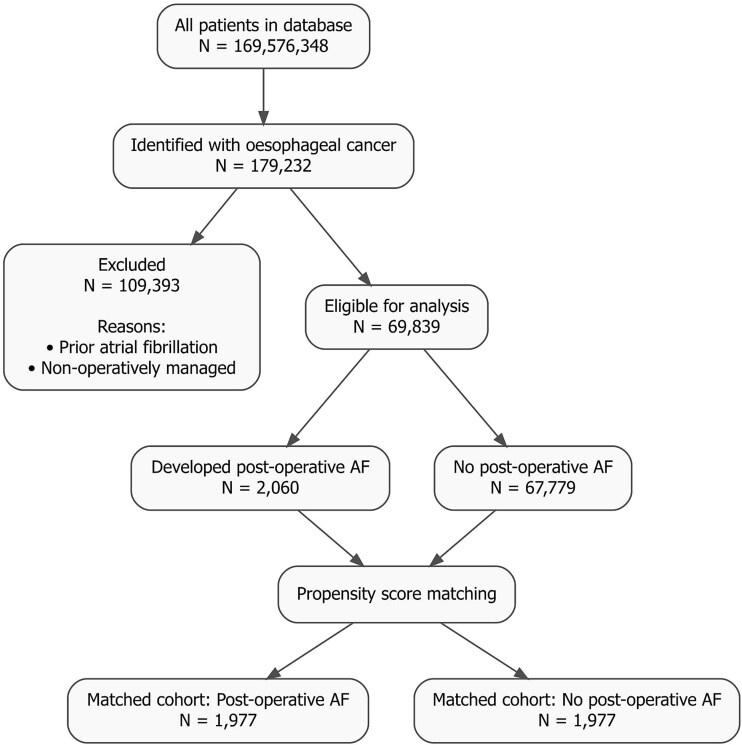
Flow diagram illustrating patient selection from the TriNetX database. Out of 169,576,348 patients, 179,232 were identified with esophageal cancer. After excluding those with a prior history of atrial fibrillation and those non-operatively managed, 69,839 patients remained eligible for analysis. Of these, 2060 patients developed postoperative atrial fibrillation, while 67,779 patients did not. Propensity score matching was performed, resulting in 1977 matched patients in each cohort.

At baseline, the study included 1977 patients in the NOAF cohort and 67,135 patients in the control cohort before PSM (Fig. 1). Before PSM, the two groups differed substantially across demographic and clinical variables.

Patients with NOAF were older than controls and had higher prevalences of major comorbidities, lifestyle, and thromboembolic risk factors (see Table 1). Overall, nearly all baseline characteristics demonstrated large standardized differences (many ≥0.20 and several >0.50), indicating substantial imbalance prior to matching (Table 1).

After PSM, 1977 NOAF patients were successfully matched 1:1 to 1977 controls with excellent covariate balance and ensuring that the two cohorts were comparable on all measured baseline characteristics (Table 1). Supplementary Fig. S1 represents our PSM matching graph demonstrating that after PSM, both cohorts were well matched.

The NOAF cohort had a mean follow-up of 286.1 ± 124 days (median 365; IQR 156), whereas the non-AF control group had a mean follow-up of 272.2 ± 137.5 days (median 365; IQR 211).

### Early (0–3 months) outcomes

NOAF was statistically associated with an increased risk of multiple adverse postoperative outcomes in the early period after surgery (Table 2). NOAF was associated with a greater observed hazard of death (HR 2.09, 95% CI 1.62–2.70; *P* < 0.001), myocardial infarction (HR 2.35, 95% CI 1.17–4.74; *P* = 0.014), heart failure (HR 2.03, 95% CI 1.32–3.11; *P* = 0.001), pneumonia (HR 1.80, 95% CI 1.42–2.29; *P* < 0.001), pneumothorax (HR 2.19, 95% CI 1.83–2.61; *P* < 0.001), PE/DVT (HR 1.76, 95% CI 1.17–2.66; *P* = 0.007), and rehospitalization (HR 2.78, 95% CI 2.42–3.20; *P* < 0.001). No significant association was observed for stroke (HR 0.98, 95% CI 0.44–2.17; *P* = 0.602). A significant association was also observed for anastomotic leak (HR 2.19, 95% CI 1.83–2.62; *P* < 0.001).

**Table 2:** Individual outcome measures comparing patients with new-onset atrial fibrillation and matched controls within 3 months following esophagectomy.

**Event**	**Outcome count (AF vs. control)**	**Hazard ratio**	**95% CI**	*P* **-value**	***E*-value**
Death	187 vs. 85	2.09	(1.62, 2.70)	<0.001	3.60
Myocardial Infarction	27 vs. 11	2.35	(1.17, 4.74)	0.014	4.14
Heart Failure	63 vs. 31	2.03	(1.32, 3.11)	0.001	3.47
PE/DVT	63 vs. 35	1.76	(1.17, 2.66)	0.007	2.92
Stroke	12 vs. 12	0.98	(0.44, 2.17)	0.602	1.16
Readmission	641 vs. 284	2.78	(2.42, 3.20)	<0.001	5.01
Anastomotic Leak	382 vs. 174	2.19	(1.83, 2.62)	<0.001	3.81
Pneumonia	182 vs. 108	1.80	(1.42, 2.29)	<0.001	3.00
Pneumothorax	352 vs. 184	2.19	(1.83, 2.61)	<0.001	3.80

Shown are outcome counts, hazard ratios (HRs), 95% confidence intervals (CIs), *P*-values, and *E*-values for postoperative outcomes. AF, atrial fibrillation; HR, hazard ratio; CI, confidence interval; PE/DVT, pulmonary embolism/deep vein thrombosis.

### Intermediate (3–12 months) outcomes

From 3 to 12 months after surgery, NOAF demonstrated a more limited pattern of associations with adverse events (Table 3). Patients with NOAF had a higher risk of death (HR 1.60, 95% CI 1.30–1.98; *P* < 0.001) and heart failure (HR 2.55, 95% CI 1.37–4.73; *P* = 0.002). No significant associations were observed for PE/DVT (HR 1.42, 95% CI 0.84–2.40; *P* = 0.184), rehospitalization (HR 1.34, 95% CI 0.76–2.37; *P* = 0.317), anastomotic leak (HR 1.82, 95% CI 0.90–3.70; *P* = 0.130), or pneumonia (HR 1.23, 95% CI 0.86–1.77; *P* = 0.252). Myocardial infarction, stroke, and pneumothorax were not included in this time-restricted analysis.

**Table 3:** Individual outcome measures comparing patients with new-onset atrial fibrillation and matched controls between 3 and 12 months following esophagectomy. Shown are patient numbers in each cohort, outcome counts, hazard ratios (HRs), 95% confidence intervals (CIs), *P*-values, and *E*-values for postoperative outcomes.

**Event**	**Outcome count (AF vs. control)**	**Hazard ratio**	**95% CI**	***P*-value**	***E*-values**
Death	227 vs. 139	1.60	(1.30, 1.98)	<0.001	2.58
Heart failure	35 vs. 14	2.55	(1.37, 4.73)	0.002	4.53
PE/DVT	34 vs. 24	1.42	(0.84, 2.40)	0.184	2.19
Rehospitalization	23 vs. 24	1.34	(0.76, 2.37)	0.317	2.02
Anastomotic leak	18 vs. 10	1.82	(0.90, 3.70)	0.13	3.04
Pneumonia	63 vs. 56	1.23	(0.86, 1.77)	0.252	1.76

Myocardial infarction, stroke, and pneumothorax were not analysed due to few outcomes being present. AF, atrial fibrillation; CI, confidence interval; HR, hazard ratio; PE/DVT, pulmonary embolism/deep vein thrombosis.

### Overall (0–12 months) results

Across the full 0–12-month postoperative period, NOAF demonstrated higher HRs for multiple outcomes compared with matched controls (Table 4). Increased hazards were observed for death (HR 1.76, 95% CI 1.50–2.07; *P* < 0.001), myocardial infarction (HR 3.25, 95% CI 1.71–6.20; *P* < 0.001), heart failure (HR 2.23, 95% CI 1.56–3.19; *P* < 0.001), PE/DVT (HR 1.95, 95% CI 1.38–2.75; *P* < 0.001), pneumonia (HR 1.51, 95% CI 1.24–1.83; *P* < 0.001), pneumothorax (HR 2.18, 95% CI 1.83–2.59; *P* < 0.001), and rehospitalization (HR 2.40, 95% CI 2.10–2.73; *P* < 0.001). A significant association was also observed for anastomotic leak (HR 2.04, 95% CI 1.75–2.38; *P* < 0.001). No significant association was observed for stroke (HR 1.31, 95% CI 0.66–2.61; *P* = 0.442).

**Table 4:** Individual outcome measures comparing patients with new-onset atrial fibrillation and matched controls within 12 months following esophagectomy. Shown are outcome counts, hazard ratios (HRs), 95% confidence intervals (CIs), *P*-values, and E-values for postoperative outcomes.

**Event**	**Outcome count (AF vs control)**	**Hazard ratio**	**95% CI**	***P*-value**	***E*-value**
Death	413 vs. 227	1.76	(1.50, 2.07)	<0.001	2.92
Myocardial Infarction	40 vs. 12	3.25	(1.71, 6.20)	<0.001	5.96
Heart Failure	98 vs. 44	2.23	(1.56, 3.19)	<0.001	3.90
PE/DVT	97 vs. 49	1.95	(1.38, 2.75)	<0.001	3.31
Stroke	19 vs. 14	1.31	(0.66, 2.61)	0.442	1.95
Readmission	664 vs. 340	2.40	(2.10, 2.73)	<0.001	4.23
Anastomotic Leak	496 vs. 248	2.04	(1.75, 2.38)	<0.001	3.5
Pneumonia	245 vs. 176	1.51	(1.24, 1.83)	<0.001	2.39
Pneumothorax	370 vs. 195	2.18	(1.83, 2.59)	<0.001	3.78

AF, atrial fibrillation; CI, confidence interval; HR, hazard ratio; PE/DVT, pulmonary embolism/deep vein thrombosis.

## DISCUSSION

**Fig. 2:**
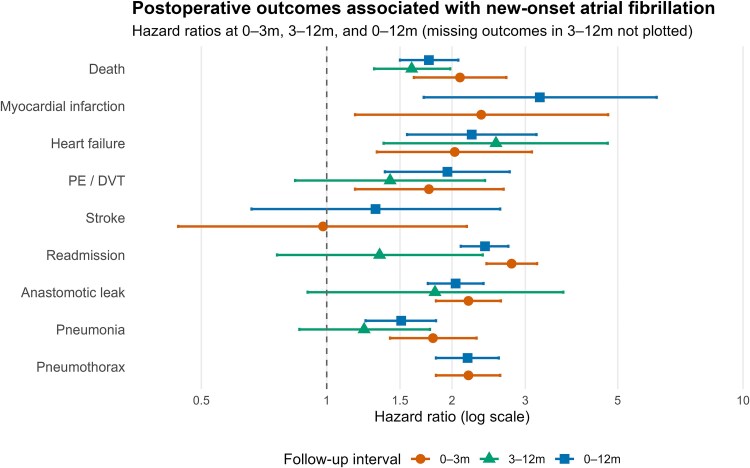
Associations between new-onset atrial fibrillation and postoperative outcomes. Forest plot displaying hazard ratios (HRs) for major adverse events among patients who developed atrial fibrillation compared with matched controls. Estimates are shown for early (0–3 months; orange), late (3–12 months; green), and overall (0–12 months; blue) follow-up. Myocardial infarction, stroke, and pneumothorax are not shown for the 3–12-month interval due to insufficient event counts.

**Fig. 3:**
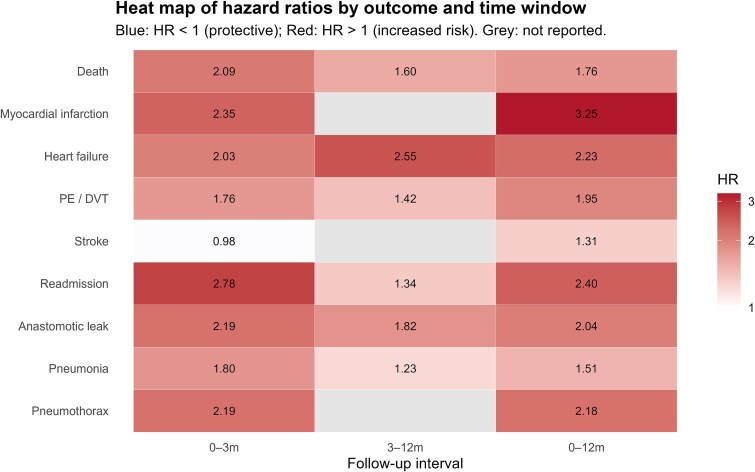
Heat map of hazard ratios (HRs) for postoperative outcomes across three follow-up intervals. Shown are HRs for new-onset atrial fibrillation compared with matched controls at 0 to 3, 3 to 12, and 0 to 12 months after surgery. Each tile displays the HR for the corresponding outcome and interval. The color scale reflects the magnitude of risk, with red indicating higher risk (HR > 1), white indicating no difference, and blue indicating lower risk (hazard ratio < 1).

In this large real-world cohort of approximately 69,000 patients undergoing esophagectomy, NOAF occurred in ~2.95% and was independently associated with increased risk of adverse outcomes. NOAF was linked to higher rates of early postoperative complications, including mortality, myocardial infarction, heart failure, respiratory complications, anastomotic leak, and rehospitalization. Although these associations attenuated after 3 months, mortality and heart failure risks remained elevated up to 12 months (Fig. 2). Figure 3 represents a heatmap of our results. These findings suggest that NOAF may represent a marker of underlying physiological stress rather than a direct causal factor, as the temporal relationship between NOAF and certain complications could not be established.

Esophagectomy is a high-risk surgical procedure, with a 90-day postoperative mortality rate of approximately 4.5% and complication rates reported in up to 59% of cases.^10^ The incidence of NOAF following esophagectomy has been reported to be notably high, potentially reflecting the esophagus’ close anatomical relationship with the left atrium and the need for pericardial manipulation during surgery. Additional contributing mechanisms include a pronounced postoperative inflammatory response, alongside established cardiovascular risk factors such as hypertension, diabetes, and vascular disease.^11^

Our findings are consistent with prior studies demonstrating an association between NOAF and adverse postoperative outcomes, although previous analyses have been limited by smaller sample sizes and heterogeneous definitions.^5^ The temporal pattern observed, with attenuation of most associations beyond 3 months but persistent excess risk of mortality and heart failure, suggests that NOAF may identify a subgroup of patients with sustained physiological vulnerability. These findings support the role of NOAF as a clinically relevant postoperative risk marker following esophagectomy and highlight the potential importance of postoperative surveillance and optimization of cardiovascular risk.

The lower observed incidence of NOAF compared with prior reports likely reflects reliance on administrative coding, which may under-detect transient or subclinical episodes.^12^ As such, the identified cohort likely represents clinically recognized and more sustained arrhythmias, introducing the possibility of selection bias and limiting generalizability to milder, self-limited AF. Therefore, the low observed incidence of NOAF compared with prior meta-analytic estimates should be considered when interpreting the results, as our findings are most generalizable to clinically recognized and coded NOAF rather than to all postoperative AF episodes detectable through systematic rhythm monitoring.

Our study aligns with previous research, demonstrating that in the short term, NOAF is associated with inflammatory complications such as pneumonia and anastomotic leakage.^13^^,^^14^ Postoperative complications, particularly pneumonia and anastomotic leak, may act as triggers for NOAF. Indeed, NOAF has been reported to occur in close temporal association with anastomotic leakage, to the extent that its onset may prompt cross-sectional imaging to investigate for this complication.^6^ Anastomotic leakage occurs in ~11.4% of esophagectomy patients,^10^ usually between day 4 and 8 postoperatively in the majority of patients, but can occur more than 2 weeks after the initial surgery.^15^^,^^16^ Smaller retrospective analyses have examined perioperative predictors of NOAF, identifying factors such as advanced age, hypertension, and respiratory complications as important risk factors.^17^ A meta-analysis also found hypertension and coronary artery disease to confer the strongest association with NOAF postoperatively, furthermore that patient with NOAF have a significant increased risk of death.^5^ NOAF has been reported in temporal proximity to anastomotic leakage, and extended monitoring has been proposed in this context for early diagnosis of complications in esophagectomy patients.^18^

Currently, there is limited evidence to support the use of prophylactic treatment for NOAF after esophagectomy. A randomized controlled trial consisting of eighty patients undergoing esophagectomy found that amiodarone prophylaxis significantly reduced the incidence of AF after transthoracic esophagectomy. Unfortunately, this was also associated with hypotension, bradycardia, and corrected QT interval prolongation, and did not result in a reduced length of critical care stay, incidence of pulmonary complications, or mortality.^19^ Future studies should aim to better characterize perioperative and patient-specific factors that increase the risk of NOAF following esophagectomy, enabling improved risk stratification. In addition, the potential role of prophylactic strategies, including pharmacological interventions, should be further evaluated to determine whether targeted or routine use in high-risk patients could reduce the incidence of this common complication and improve postoperative outcome.

### Limitations

Our findings should be interpreted considering several limitations. First, as an observational analysis, this study can identify associations but cannot establish causality. Second, the retrospective design means that data were not randomized or experimentally controlled. Although PSM was applied to minimize confounding, residual bias may persist due to incomplete or inaccurate recording within electronic health records. Third, TriNetX data are derived from administrative and diagnostic coding systems primarily intended for clinical documentation and billing, rather than for research standardization. As such, we were unable to verify coding accuracy through direct review of individual medical records, and some diagnostic or procedural information may have been under- or misclassified. Importantly, the database lacks reliable granularity for certain operative and oncologic variables. Two clinically important determinants that were not consistently available include surgical approach and detailed. Although a large number of patients were coded broadly under ‘esophagectomy,’ approach-specific procedural codes (e.g. Ivor Lewis or transhiatal techniques) were infrequently and inconsistently captured, rendering subgroup analyses by surgical technique statistically underpowered and susceptible to misclassification. Similarly, detailed variables such as anastomotic location, tumor stage, neoadjuvant therapy regimens, and precise perioperative management strategies were not consistently available. Although *E*-values were calculated to contextualize the potential impact of unmeasured confounding, they do not exclude residual confounding from unavailable operative, oncological, or perioperative factors, and the observed associations should therefore be interpreted cautiously. Temporal precision of NOAF onset relative to surgery is also limited by the structure of administrative timestamp data. The absence of granular event timestamps in TriNetX precluded determination of temporal ordering between NOAF and certain complications (e.g. anastomotic leak, pneumonia), limiting our ability to exclude reverse causation. Importantly, anastomotic leakage is a predominantly early postoperative complication. In our dataset, most leak events occurred within the first 3 months after surgery, consistent with expected biological timing. Events coded beyond this window likely reflect ongoing management or delayed documentation rather than true late de novo leak formation. As such, in our results, NOAF may represent a marker of postoperative clinical deterioration rather than an independent explanatory factor for mortality. This may be associated with both the occurrence of NOAF and postoperative outcome. These factors collectively limit causal interpretation and suggest that our results should be considered hypothesis-generating rather than confirmatory in nature.

## CONCLUSION

NOAF after esophagectomy is associated with adverse postoperative outcomes, including increased mortality, heart failure, and rehospitalization, with effects persisting up to 12 months. The strong association with anastomotic leakage highlights its potential role as an early marker of postoperative complications and supports the need for targeted monitoring and management.

## Synopsis

This study examines the association between new-onset atrial fibrillation after esophagectomy and short- and long-term cardiovascular and postoperative outcomes using a large international real-world database with propensity score–matched analysis.

## Supplementary Material

Supplementary_figure_1_doag058

## ABBREVIATIONS

*CI*, confidence interval; *CPT*, current procedural terminology; *GDPR*, General Data Protection Regulation; *HCO*, Healthcare Organization; *HIPAA*, Health Insurance Portability and Accountability Act; *HR*, hazard ratio; *ICD-10*, International Classification of Diseases, Tenth Revision; *IQR*, interquartile range; *KM*, Kaplan–Meier; *MI*, myocardial infarction; *PSM*, propensity score matching; *RR*, risk ratio; *SD*, standard deviation; *SNOMED*, Systematized Nomenclature of Medicine—Clinical Terms; *SSMD*, strictly standardized mean difference; *TriNetX*, TriNetX Global Health Research Network; *VTE*, venous thromboembolism

## Notes

Specific author contributions: Hani Essa (Conceptualization, Data curation, Formal analysis, Investigation, Methodology, Project administration, Writing—original draft, Writing—review & editing), Ashwin Balu (Conceptualization, Methodology, Writing—original draft), Brian Johnston (Writing—review & editing), Nathan Stephens (Writing—review & editing), Gregory Y. Lip (Supervision, Writing—review & editing), and Ingeborg D. Welters (Supervision, Writing—review & editing)

Financial disclosure: The authors have no financial disclosures to make. There were no conflicts of interest

Financial support: None.

Potential competing interests: None.
