# Supplementary figures and images for: Impact of new-onset atrial fibrillation on 12-month outcomes and mortality after esophagectomy
Source: Dis Esophagus. 2026 Jun 22;39(3):doag058. doi: 10.1093/dote/doag058 (PMC13284774; doi:10.1093/dote/doag058)

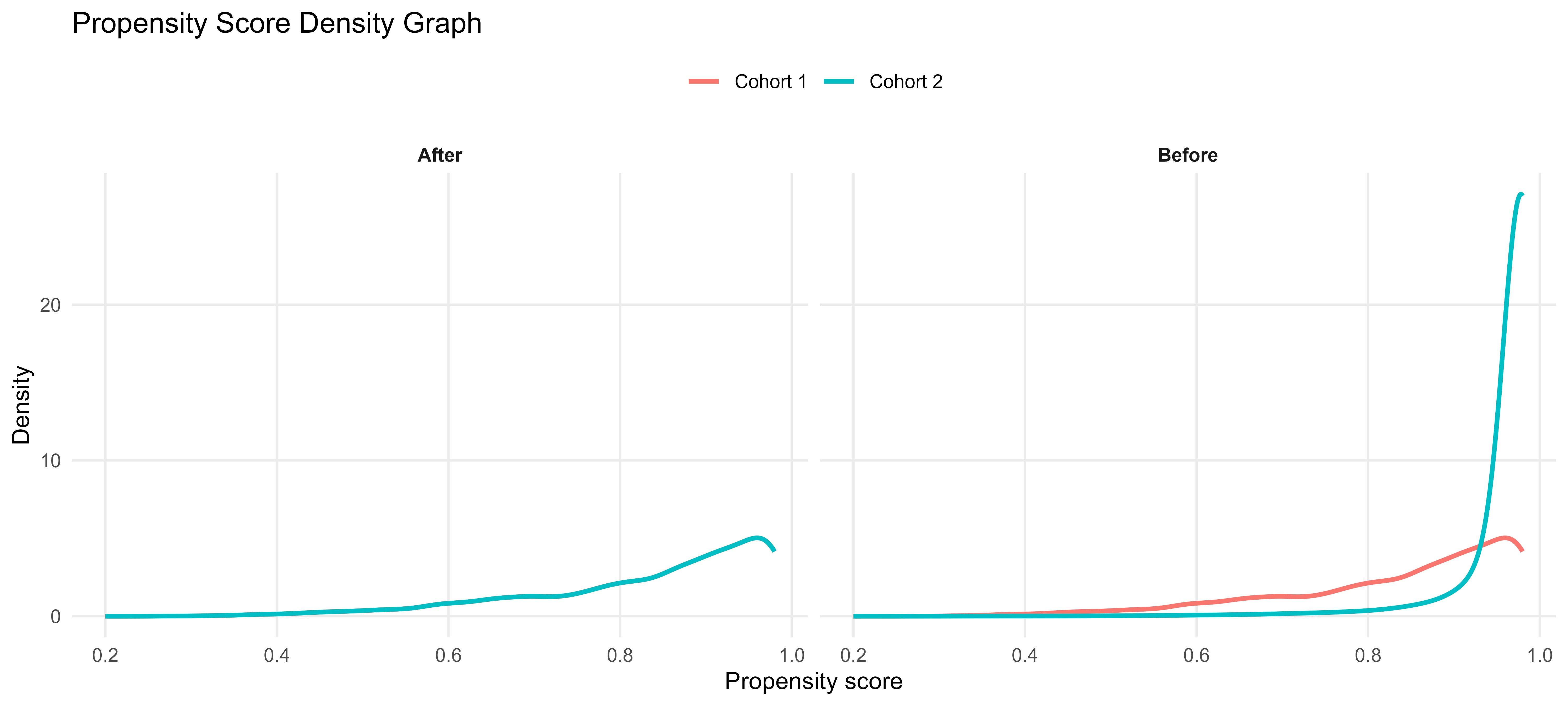

Supplement: Supplementary_figure_1_doag058 [file supplementary_figure_1_doag058.jpeg]
